# How Do We Segment Text? Two-Stage Chunking Operation in Reading

**DOI:** 10.1523/ENEURO.0425-19.2020

**Published:** 2020-06-05

**Authors:** Jinbiao Yang (杨金骉), Qing Cai (蔡清), Xing Tian (田兴)

**Affiliations:** 1 Key Laboratory of Brain Functional Genomics– (Ministry of Education & Science and Technology Commission of Shanghai Municipality), Affiliated Mental Health Center, ECNU Shanghai Changning Mental Health Center, School of Psychology and Cognitive Science, East China Normal University, Shanghai 200062, China; 2 NYU-ECNU Institute of Brain and Cognitive Science at New York University Shanghai, Shanghai 200062, China; 3 Division of Arts and Sciences, New York University Shanghai, Shanghai 200122, China; 4 Max Planck Institute for Psycholinguistics, 6525 XD Nijmegen, The Netherlands; 5 Centre for Language Studies, Radboud University, 6500 HD Nijmegen, The Netherlands

**Keywords:** EEG, hierarchy, lexical access, reading, segmentation

## Abstract

Chunking in language comprehension is a process that segments continuous linguistic input into smaller chunks that are in the reader’s mental lexicon. Effective chunking during reading facilitates disambiguation and enhances efficiency for comprehension. However, the chunking mechanisms remain elusive, especially in reading, given that information arrives simultaneously yet the written systems may not have explicit cues for labeling boundaries such as Chinese. What are the mechanisms of chunking that mediates the reading of the text that contains hierarchical information? We investigated this question by manipulating the lexical status of the chunks at distinct levels in four-character Chinese strings, including the two-character local chunk and four-character global chunk. Male and female human participants were asked to make lexical decisions on these strings in a behavioral experiment, followed by a passive reading task when their electroencephalography (EEG) was recorded. The behavioral results showed that the lexical decision time of lexicalized two-character local chunks was influenced by the lexical status of the four-character global chunk, but not vice versa, which indicated the processing of global chunks possessed priority over the local chunks. The EEG results revealed that familiar lexical chunks were detected simultaneously at both levels and further processed in a different temporal order, the onset of lexical access for the global chunks was earlier than that of local chunks. These consistent results suggest a two-stage operation for chunking in reading, the simultaneous detection of familiar lexical chunks at multiple levels around 100 ms followed by recognition of chunks with global precedence.

## Significance Statement

The learners of a new language often read word by word. However, why can proficient readers read multiple words at a time? The current study investigates how we efficiently segment a complicated text into smaller pieces and how we process these pieces. Participants read Chinese strings with different structures while their key-press responses and brain electroencephalography (EEG) signals were recorded. We found that texts were quickly (∼100 ms from their occurrences) segmented to varied sizes of pieces, and larger pieces were then processed earlier than small pieces. Our results suggest that readers can use existing knowledge to efficiently segment and process written information.

## Introduction

Reading is arguably one of the unique human intelligences. However, how we process written texts remains elusive. For instance, how can we comprehend a complex sentence? A sentence consists of many letters/characters that form a hierarchical structure of text chunks (e.g., morphemes, words, and phrases). Readers need to incrementally segment a complex sentence into smaller chunks that map onto their mental lexicon. This process is termed as text chunking (Reali and Christiansen, 2007; Gobet et al., 2016). What are the small chunks during chunking? How do we process the chunks? To answer those questions, this study investigated the cognitive procedure of text chunking.

Words and their sublevel (morphemes) are usually assumed as the basic units in reading models in psycholinguistics and computer science (McClelland and Rumelhart, 1981a; Coltheart et al., 2001; Taft, 2013). However, eye-tracking studies suggested we can perceive the text information longer than a word at one time (Rayner, 1998). Our working memory also allows us to remember familiar multiple words (Miller, 1956). Even more, multiword expressions can be stored in our mental lexicons (Arnon and Snider, 2010; Siyanova-Chanturia et al., 2017). These studies suggest the multiword representations and the beyond-word processing are feasible. Moreover, relying on larger chunks effectively reduces the cognitive load while processing sentences: fewer chunks to be interpreted and integrated (Ellis, 2003; Krishnamurthy, 2003; Blache and Rauzy, 2012). Furthermore, the semantic combination of constituents can be different from holistic meaning (Goldberg, 1995). One extreme example is idioms, as the metaphors of an idiom can be distinct from their literal meanings of smaller constituents. Multiword representation is required in certain contexts to avoid ambiguity. Therefore, multiword chunks, as well as word chunks, could be the units during chunking.

What is the relation between the processes of word chunks and multiword chunks during chunking? The studies of compound words (a single lexical entity but consists of more than one root morphemes, e.g., “flagship”) may offer hints. According to the dual-route models of compound-word processing, both the whole word and its constituents are processed at the same time or are selected to process each level flexibly (Andrews et al., 2004; Koester et al., 2007; MacGregor and Shtyrov, 2013; Semenza and Luzzatti, 2014; Blache, 2015). In a similar vein, we hypothesized that all the familiar lexical chunks, no matter which level it is, could be processed simultaneously. More specifically, the detection of chunks would be the first step in chunking, and the detection of chunks at multiple levels would occur at the same time, as the early lexical familiarity checking assumed in the E-Z reader model (Reichle et al., 2003).

How does the multilevel operation unfold in the chunking process? Which level has the priority after being detected? The word superiority effect indicates that the recognition of letters within words is better than letters in nonwords or stand-alone letters (Reicher, 1969). It suggests that the word has priority over the letter in reading. Similarly, the processing priority of global chunks can reduce the steps of integration and avoid the ambiguity to enhance the efficiency of language processing (Ellis, 2003; Krishnamurthy, 2003; Blache and Rauzy, 2012). Generalizing from the word superiority effect, we hypothesized that global chunks took priority over the parts and would be initiated first in the processing stage after detection.

In this study, we used Chinese four-character strings to investigate the chunking operation in reading. Chinese written system is an ideal model for observing multilevel chunking because the Chinese do not have explicit word boundaries. Each Chinese character is a basic lexical unit with a similar length. Four characters can form two levels of chunks, chunks with two characters (hereafter as the local level chunks) and a chunk with four characters (hereafter as the global level chunk). The lexicality was manipulated at both levels so that four types of stimuli were included (phrase, idiom, random words, and random characters). In the behavioral experiment, we investigated the interaction between the global and local chunks in reading by a lexical decision task at different levels of chunks. Moreover, an electroencephalography (EEG) experiment was conducted to investigate the temporal dynamics of detection and recognition stages in the multilevel chunking operation.

## Materials and Methods

### Participants

Twenty-one healthy native Chinese speakers (10 males, mean age 21 years, range 18–30 years) with normal or corrected-normal vision participated in both behavior and EEG experiments for financial compensation. Five participants who produced extensive EEG artifacts were excluded from EEG analysis. Hence, a total of 16 participants were included in the EEG study. The experiments were approved by the Research Ethics Committee of East China Normal University and New York University Shanghai. Written informed consents were obtained from all participants before the experiments.

### Stimuli

All stimuli are four-Chinese-character strings. Two factors are included when designing these stimuli. The first factor is the chunk size that contains two levels, a global size of four characters and a local size of two characters. The second factor is lexicality (word or nonword) at each chunk size. These two factors are fully crossed and yield four types of stimuli. We denote chunk size using upper case letters, G for global and L for local, and use lower case letters for lexicality in each chunk size (w for word and n for nonword). For example, *GnLw* stands for the condition of stimuli that are four-character nonwords at the global level made of two two-character words at the local level. Note that the stimuli in *GwLn* are Chinese idioms, “Chengyu.” They are lexicalized compound phrases that consist of four independent mono-morphemic characters. None of the two characters in Chengyu can form a common word, whereas the four characters together form an idiomatic expression. Chengyu generally expresses the gist or moral message of myths, stories, or historical events from which they were derived. Therefore, the meaning of a Chengyu usually surpasses the sum of the meanings from the four characters. The four types of stimuli are listed in Table 1.

**Table 1:** Stimuli description

	Local word	Local nonword
Global word	*GwLw*: lexicalized compound phrase composed of two two-character words. E.g., “*希 腊 神 话* ” (pinyin: xī là shén huà), translation: Greek mythologies. “*希 腊* ” and “*神 话* ” means “Greek” and “mythologies” in Chinese, respectively	*GwLn*: lexicalized compound phrases that consist of 4 independent mono-morphemic characters (Chinese idioms “Chengyu”). E.g., “*以 逸 待 劳* ” (pinyin: yǐ yì dài láo), translation: wait for the exhausted enemy at your ease. “*以 逸* ” and “*待 劳* ” are not words in Chinese
Global nonword	*GnLw*: non-lexicalized compound phrase composed of two two-character words. E.g., “*存 款 电 脑* ” (pinyin: cún kuǎn diàn nǎo), translation: deposit-computer. “*存 款* ” and “*电 脑* ” means “deposit” and “computer” in Chinese, respectively	*GnLn*: random character string, nonwords at both levels. E.g., “*投 其 顾 此* ” (pinyin: tóu qí gù cǐ), a nonsense phrase. “*投 其* ” and “*顾 此* ” are not words in Chinese either

We selected and created all stimuli with the following steps. We extracted the *GwLw* and *GwLn* stimuli from a database of Sogou Pinyin (https://www.sogou.com/labs/resource/w.php) and a database of Chinese characters (CharDB: data version: 0.98.1; program version: 0.97.2; https://chardb.cls.ru.nl/). All the *GwLw* and *GwLn* stimuli satisfied the following criteria at the global level: (1) noun (the part of speech is determined by a record of the lexicon of Jieba v0.36: https://github.com/fxsjy/jieba); (2) high-frequency (the frequency was determined by the database of the Sogou Pinyin, and the high-frequency meant the frequency above 3000); and (3) no duplicative characters (e.g., “高高兴兴,” translation: happy). Moreover, the *GwLw* stimuli satisfied the following criteria at the local level: (1) both two-character words were nouns, and (2) high-frequency words. Moreover, the lexicality of *GwLn* stimuli at the local level was verified by checking if the first two or last two characters’ combination did not exist in the Sogou Pinyin database. These selection criteria made the *GwLw* and *GwLn* stimuli consistent in all aspects except the lexical status at the local level.

The *GnLw* stimuli were created by randomly pairing two different two-character words, the Lw in GnLw and GwLw follow the same criteria. So the only difference between the *GwLw* and *GnLw* was the lexical status at the global level. Finally, the *GnLn* stimuli were created by randomly mixing four different characters, and none of the first or last two characters’ combinations existed in the Sogou Pinyin database. Characters used in all stimuli have log frequency ranging from 3.011 to 5.344, with stroke counts ranging from 4 to 13. The character’s log frequency was determined by the Subtitle Database (Cai and Brysbaert, 2010).

The distinction between word and nonword was further controlled by familiarity. Twelve participants who were not in the main experiment were asked to rate the familiarities of either the entire four-character or the constituents of two-character strings as being words or not. The rating range was from 1 to 5, where 1 stands for unfamiliar strings/nonwords and 5 for familiar words. The strings that were rated from 2 to 4 were removed and remained the stimuli that were either very familiar words or very unfamiliar nonwords in a pool. Eighty stimuli in each condition were randomly selected from the pool and used in this study.

### Procedure behavioral experiment

In each trial, participants were first asked to focus on a cross presented at the center of the screen. After 400 ms, the fixation cross disappeared, and a four-character string was shown until response. A line also appeared either under the entire four-character string or under the two-character string (the first or the last two characters). Participants were asked to make a lexical decision about the underlined string, either the entire string (global task henceforth) or the constitute of first or last two-character string (local task henceforth) by pressing either “F” or “J” on the keyboard as fast as possible. Participants had a maximum of 3 s to respond. Responses and reaction time were collected. Response keys were counterbalanced across participants. The intertrial intervals were randomly selected from a range from 800 to 1000 ms.

Four stimuli types (*GwLw*, *GwLn*, *GnLw*, and *GnLn*) were fully crossed with task types (global task vs local task) and yielded eight conditions; 320 trials were included in this experiment. Half of the trials were randomly selected and used in the global task and the other half in the local task. The order of conditions was randomized. The experimental presentation was programmed on a Python package, Expy, which is a software for presenting and controlling psychological experiments (https://expy.readthedocs.io/).

#### Behavioral data analysis

All participants had response accuracy exceeding 85%, and the average accuracy was 92%. No participant’s data were excluded. Trials with error responses were removed before analysis. We applied repeated measures three-way ANOVA (analysis of variance) on the reaction time data with factors of global-level lexicality, local-level lexicality, and task, followed by planned *t* tests for testing specific hypotheses.

### Procedure EEG experiment

The same group of subjects participated in the EEG experiment. The EEG experiment shared the same stimuli list with the behavioral experiment, but both the procedure and the task are different. First, the display of each character string lasted for 300 ms. Participants were asked to read the underlined parts of the stimuli (to keep their attention on the stimuli), but they did not perform any lexical decision task. We used all 320 strings with 80 for each stimuli type in the global task and repeated once in the local task. Moreover, 320 four-symbol strings were included as the visual baseline in the EEG experiment. The symbols in a symbol string trial were randomly sampled with replacement from four symbols (□, △, ◇, and ○). Underlines were included in the symbol trials similar to those in the global and local tasks in experimental trials. Half of the trials were randomly selected and used in the global task and the other half in the local task. To guarantee participants’ attention on the stimuli, we randomly inserted strings of digits for 100 ms, and participants were asked to report the underlined digits by pressing number buttons on a keyboard. About 48 number-report trials were presented to each participant.

#### EEG recording

EEG signals were recorded with a 32-electrode active electrodes system (actiChamp system, Brain Products GmbH). FP1 and FP2 were used to monitor vertical eye movements. Electrode impedances were kept below 10 kΩ. Data were continuously recorded in single DC mode. Data were sampled at 500 Hz, online referenced to the Cz.

#### EEG data analyses

EEG data were preprocessed using EEGLAB (version 13.5.4b; Delorme and Makeig, 2004). Data were bandpass filtered (0.1–30 Hz, Hamming windowed sinc FIR filter), and re-referenced to the average reference. The preprocessed data were epoched between –200 and 800 ms relative to the onset of strings and baseline-corrected using the 200-ms prestimulus period. The trials with eye blinks were rejected if the amplitude within the 1000-ms epoch exceeded ±50 μV. Remaining trials with apparent noise were rejected manually. Approximately 15% of trials were rejected. Five participants who produced a large number of artifacts or showed continuous α waves were excluded from further analysis. Epochs in each condition were averaged and created an event-related potentials (ERPs). Root mean square (RMS) responses were also calculated as a geometric mean of all channels.

In addition to the univariate analyses on ERPs and RMS responses, our analysis used the topographic patterns or distributions across all sensors rather than the response amplitude in selected groups of sensors, as it can provide more holistic and unbiased information. Such multivariate methods can collectively reflect spatial and temporal information and offer more power to test psychological and neuroscience hypothesis by overcoming problems such as individual differences, sensor selection and reference selection in EEG (Murray et al., 2008; Tian and Huber, 2008; Tian et al., 2011; Yang et al., 2018; Wang et al., 2019). Two multivariate-based methods [clustering and topographic ANOVA (TANOVA)] and one mass-univariate method (analysis of the topographic distribution of amplitude differences) were applied as following.

##### Clustering

A clustering method on the ERP topographic responses was implemented first. This unsupervised machine learning method groups data across all conditions by forming temporal clusters based on the similarity of their topographical patterns. This clustering method is a data-driven method, in which it explores the pattern similarity in topographies in all conditions. The clustering algorithm organizes data at different time points into distinct clusters, so that we can explore the temporal dynamics of pattern changes. Moreover, if one considers the clusters reflecting different processing stages, this analysis can identify the processing stages in each condition and display the temporal differences of any specific stage among conditions. We used K-means, the most popular algorithm for clustering. The clustering analysis is an omnibus test about neural dynamics, which can detect clusters and set up the time windows of interest for the following analyses.

The procedure of clustering algorithm covers three steps: (1) averaged EEG data across all participants to get ERPs at each time point for each condition; (2) defined ERPs at each time point in each condition as a sample, and the amplitudes of 32 electrodes were used as features in a sample; (3) K-means algorithm was conducted at all samples. The K-means algorithm is data driven. The target cluster number (K) can range from the minimum of one to the number of data points. We assumed that the baseline period to a fixation involved rest or random cognitive processes. The topographies would be consistent random patterns that were different from later sequences of topographies induced by stimuli. If the K-means separated the baseline period into more than one cluster, it was most likely overfitting. Therefore, we set the criterion of getting two clusters at the baseline period as a stopping point for increasing the number of clusters. That is, the K-means algorithm was conducted at all samples. The number of clusters was initially two and increased until the clustering result included more than one cluster at the baseline stage.

##### Analysis of the topographic distribution of amplitude differences

We calculated the response amplitudes across all sensors at a given time window. The changes in the amplitude differences can reflect the processing dynamics. Especially, the earliest time point that shows significant differences would indicate the temporal onset of interest. Moreover, the spatial extent of experimental effects can be estimated by the distribution of sensors in which the amplitude differences are observed. At a window size of 20 ms, we checked the significant electrodes (Yang et al., 2018) to obtain the distribution differences between conditions. Because there were 32 comparisons in each time window, the *p* values were corrected by false discovery rate (FDR). The primary purpose of this analysis was to identify the possible onset timing of any amplitude differences between conditions. However, the power of detecting the onset using a measure of amplitude could be small. To reduce the Type II error, we did not apply corrections across time so that we can reduce the chance of missing the exact onset time of amplitude differences.

##### TANOVA

We further investigate the patterns of topographies by considering all sensors at the same time to infer the differences in underlying neural processes across conditions. A single index was calculated to indicate topographic information. Mathematically, each topography can be viewed as an *n*-dimensional vector, where the *n* equals the number of sensors. The divergence between the topographies of two experimental conditions can be quantified by the cosine value of the high-dimensional angle between two vectors (Tian and Huber, 2008). The cosine distance has a range from 0 to 2, where 0 stands for identical topographies and 2 exactly opposite patterns. Note that the cosine distance represents the similarity between the response patterns in topographies and is free from the difference of response magnitude because the measure of cosine distance is normalized by the vector length. To statistically test the cosine distance between topographies and to infer the underlying neural processing in different conditions, we applied an algorithm named TANOVA (Murray et al., 2008; Tian and Huber, 2008; Brunet et al., 2011; Tian et al., 2011; Lange et al., 2015). In TANOVA, null hypothesis distribution is generated by shuffling the condition labels, and we here shuffled the condition labels on the subjects’ ERPs using the EasyEEG toolbox (Yang et al., 2018; strategy 2, shuffle times: 1000, window size: 10 ms). Furthermore, the temporal clusters in the TANOVA results were identified by a precluster threshold of 0.1 and were tested by the cluster-based permutation with the corrected threshold of 0.05 (Maris and Oostenveld, 2007).

## Results

### Behavioral experimental results

Reaction time was subject to repeated measures three-way ANOVA with the factors of global-level lexicality, local-level lexicality, and task. The main effect of global-level lexicality was significant (*F*_(1,20)_ = 10.83, *p *<* *0.01), suggesting that participants took longer time to identify global-level nonwords than global-level words. The main effect of local-level lexicality is significant (*F*_(1,20)_ = 6.30, *p *=* *0.02), suggesting participants took longer time to identify local-level nonwords than local-level words. However, the main effect of task is not significant (*F*_(1,20)_ = 1.07, *p *=* *0.31), suggesting different tasks that require participants to respond to either global or local chunks have a similar level of difficulty. More importantly, all three two-way interactions are significant, global-level lexicality × local-level lexicality (*F*_(1,20)_ = 57.11, *p *<* *0.001), global-level lexicality × task (*F*_(1,20)_ = 16.02, *p *<* *0.001), and local-level lexicality × task (*F*_(1,20)_ = 57.11, *p *<* *0.001).

**Figure 1.**
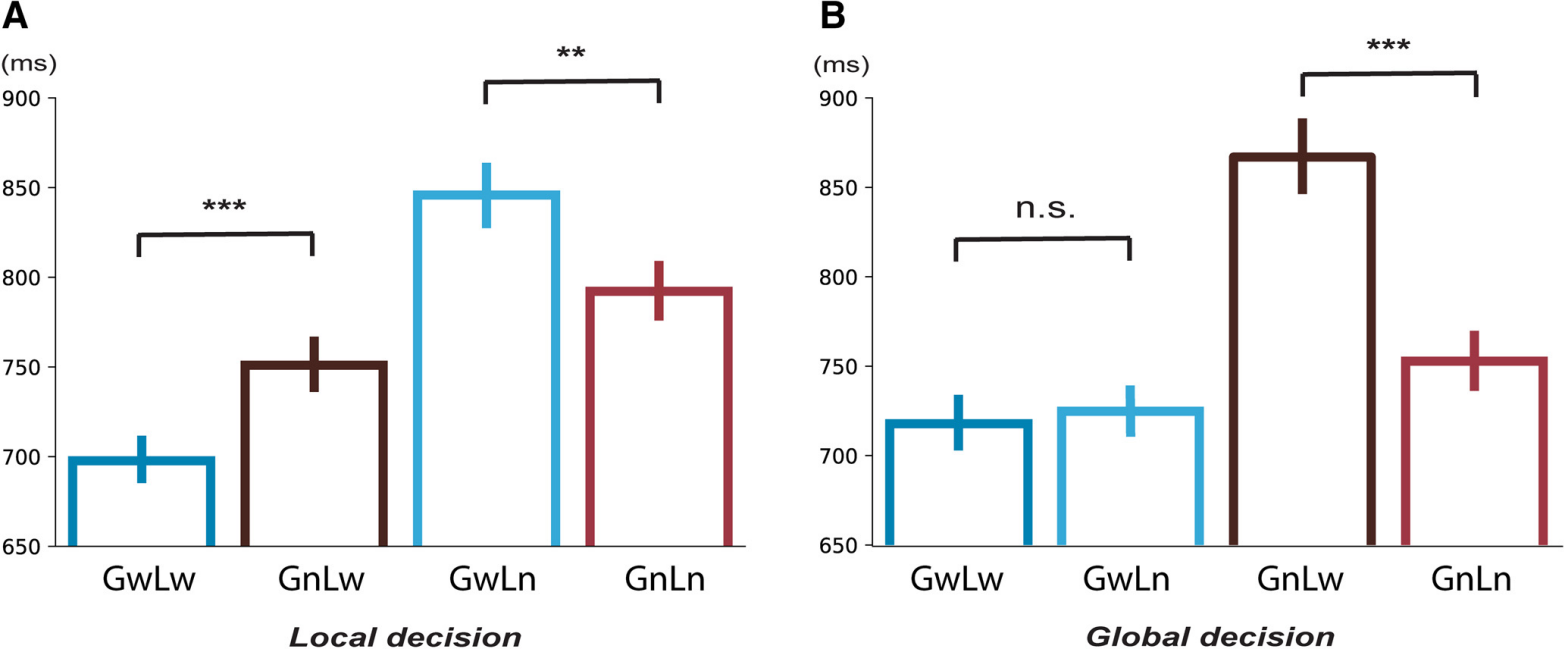
Results of the behavioral experiment. ***A***, ***B***, Reaction time results in the global and local tasks, respectively. In each plot, condition labels are provided along the *x*-axis. Error bars represent ±1 SEM (standard error of the mean). Each planned paired test was represented by the line linking two bars; n.s., not significant; **p <* *0.01, ***p <* *0.001.

Planned *post hoc t* tests were further conducted in each factor to specify the observed significant interactions. First, we examined how global information affect processing at the local level (Fig. 1*A*). In the local task, the reaction time in *GnLw* was significantly longer than that in *GwLw* (*t*_(20)_ = 5.7145, *p *<* *0.001, difference = 55 ms), suggesting that the nonwords at the global level significantly slowed down the lexical decision of words at the local level. Moreover, the reaction time of *GwLn* was significantly longer than *GnLn* in the local task (*t*_(20)_ = 3.6214, *p *=* *0.002, difference = 54 ms), suggesting that the words at the global level also slowed down the lexical decision of nonwords at the local level. Second, we examined how the local chunk could affect processing at the global level (Fig. 1*B*). In the global task, we did not find a significant difference between reaction time to *GwLw* and *GwLn* (*t*_(20)_ = −1.0091, *p *=* *0.32; Fig. 1*B*, left), suggesting that the lexical status of local chunks cannot affect the lexical decision of words at the global chunks. These results collaboratively suggest that processing at the global level may take priority.

Another comparison on how the local chunk could affect processing at the global level revealed that the reaction time of *GnLw* was significantly longer than that of *GnLn* in the global task (*t*_(20)_ = 6.6874, *p *<* *0.001, difference = 112 ms; Fig. 1*B*, right). This result suggests that the decisions of global chunks and local chunks could be parallel when the global decision took too long. The lexical information at the local level may leak through to the processing of global chunks and influence the decision of nonwords. The behavioral results demonstrate a unidirectional influence from the global level to the local level when the task targets are words and interactions between levels when the task targets are nonwords that need more time to make decisions. That is, global chunks may take priority in lexical processing. Whereas the processing of global and local chunks could be parallel when global decision took too long, the lexical information at one level may leak through to the process of the other level and influence the decision of nonwords. We further test the processing dynamics in an EEG experiment.

To examine and exclude the possible effects of the underline position, we extracted the trials with two-character underlines and ran a repeated measures three-way ANOVA with the factors of global-level lexicality, local-level lexicality, and underline position. The underline position showed neither main effect (*F*_(1,20)_ = 1.61, *p *=* *0.22), nor interaction effect with global-level lexicality (*F*_(1,20)_ = 0.01, *p *= 0.92), nor interaction effect with local-level lexicality (*F*_(1,20)_ = 0.38, *p *=* *0.54). These results suggest that positions of stimuli that were relevant to the task did not affect response speed.

### EEG results

#### Clustering revealed distinct stages of chunking

**Figure 2.**
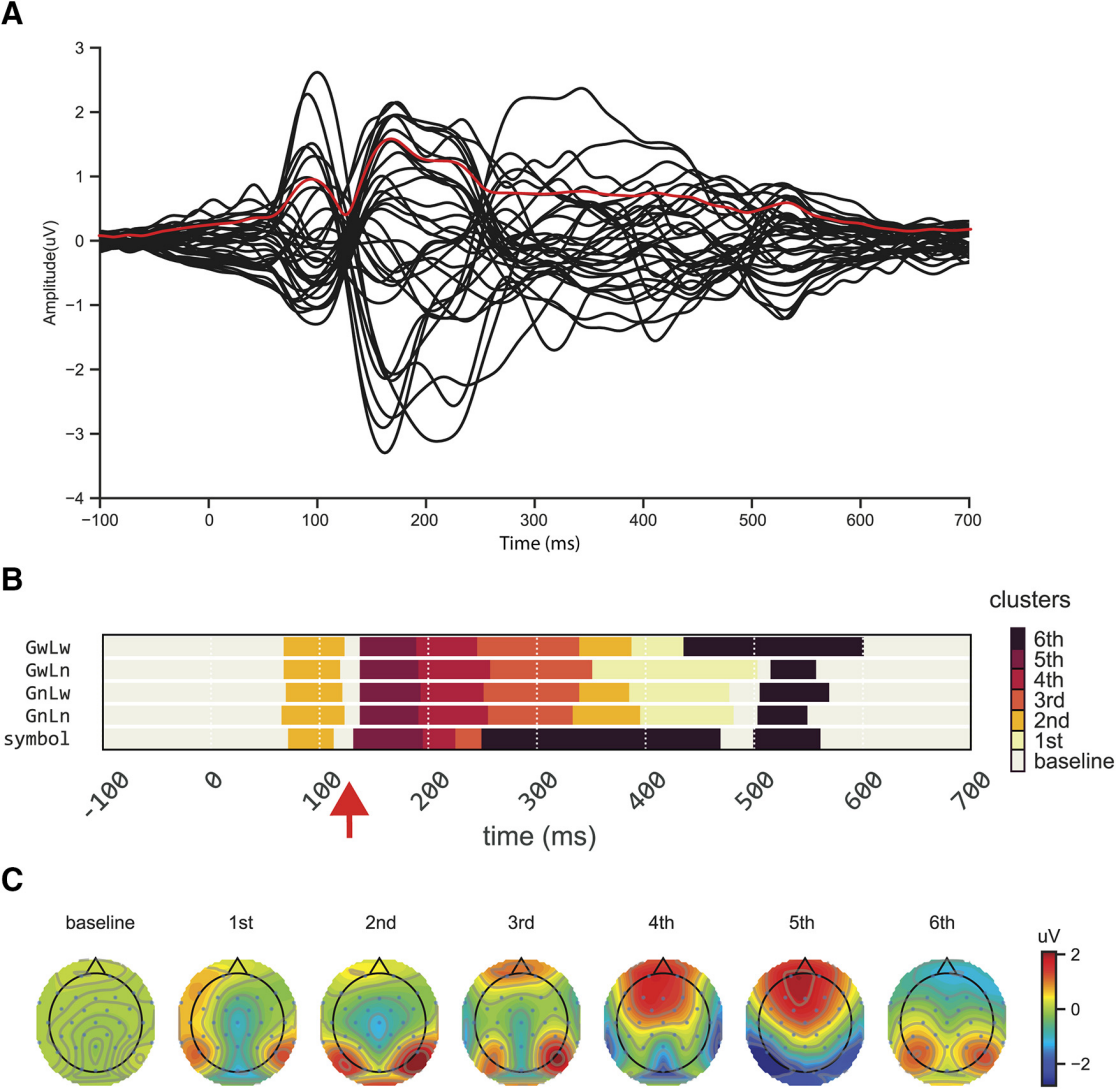
The dynamics of ERP responses and clustering results. ***A***, Averaged ERPs waveform responses of all conditions from 32 electrodes (black lines), and RMS waveform response across all electrodes (red line). ***B***, Temporal clustering results of topographies for four conditions (*GwLw*, *GwLn*, *GnLw*, and *GnLn*) and a baseline symbol condition (symbol). Different colors represent distinct clusters. Samples in the same color but at different time points indicate that they are grouped into the same cluster, sharing similar features but occurring at different times. The temperature of colors represents the rank of the cluster distance relative to the cluster baseline (cluster defined by the baseline period). Approximately 80 ms after stimulus onset, a novel cluster (the second cluster) appears at the same time across five conditions, followed by another new cluster. However, in the symbol condition, the third cluster (∼250 ms) appears earlier with a much shorter duration than four-character string conditions. ***C***, The topographies of each cluster.

We first conducted the clustering analysis to explore the dynamics of ERP responses. The clustering algorithm aimed to separate the continuous ERP responses into distinct stages based on common features observed across time. As shown in Figure 2, the clustering results were reliable as similar clusters were observed continuously in each condition.

More importantly, clear temporal profiles were revealed by the clustering analysis in all conditions. First, the same cluster was observed in the baseline period until around 80 ms after stimulus onset, as well as at the end of epochs (∼600 ms after onset) among all types of stimuli. The clustering in these periods was presumably because few cognitive processes that relate to the stimuli or task were available or manifested in the ERP topographies. Second, a novel cluster (the second cluster) appeared after 80 ms across five conditions. The clustering spanned in similar latencies as N1/P2 components, presumably reflecting visual processing. However, different dynamics was observed across conditions after 200 ms. The third cluster appeared earlier in the symbol condition with a much shorter duration than the four experimental string conditions in which the third cluster appeared around 250 ms and lasted ∼90 ms. Moreover, in the symbol condition, the third cluster was immediately followed by the sixth cluster that did not appear until 500 ms in the four experimental string conditions (except in *GwLw* condition around 430 ms). The early start and long-lasting sixth cluster in the symbol condition was accompanied by the missing of the first and second clusters that appeared around 320 ms and lasted till 450 ms in other conditions.

More interestingly, a 15-ms period around 130 ms was labeled as the cluster baseline that was grouped in the baseline and the end of epoch periods. This formed a short gap that broke the early processing into two stages. The clustering results set up the time windows of interest for the following analyses. We focused on the components in early timing to further investigate the underlying processes of chunking operation.

#### Chunk detection in the earliest stage

**Figure 3.**
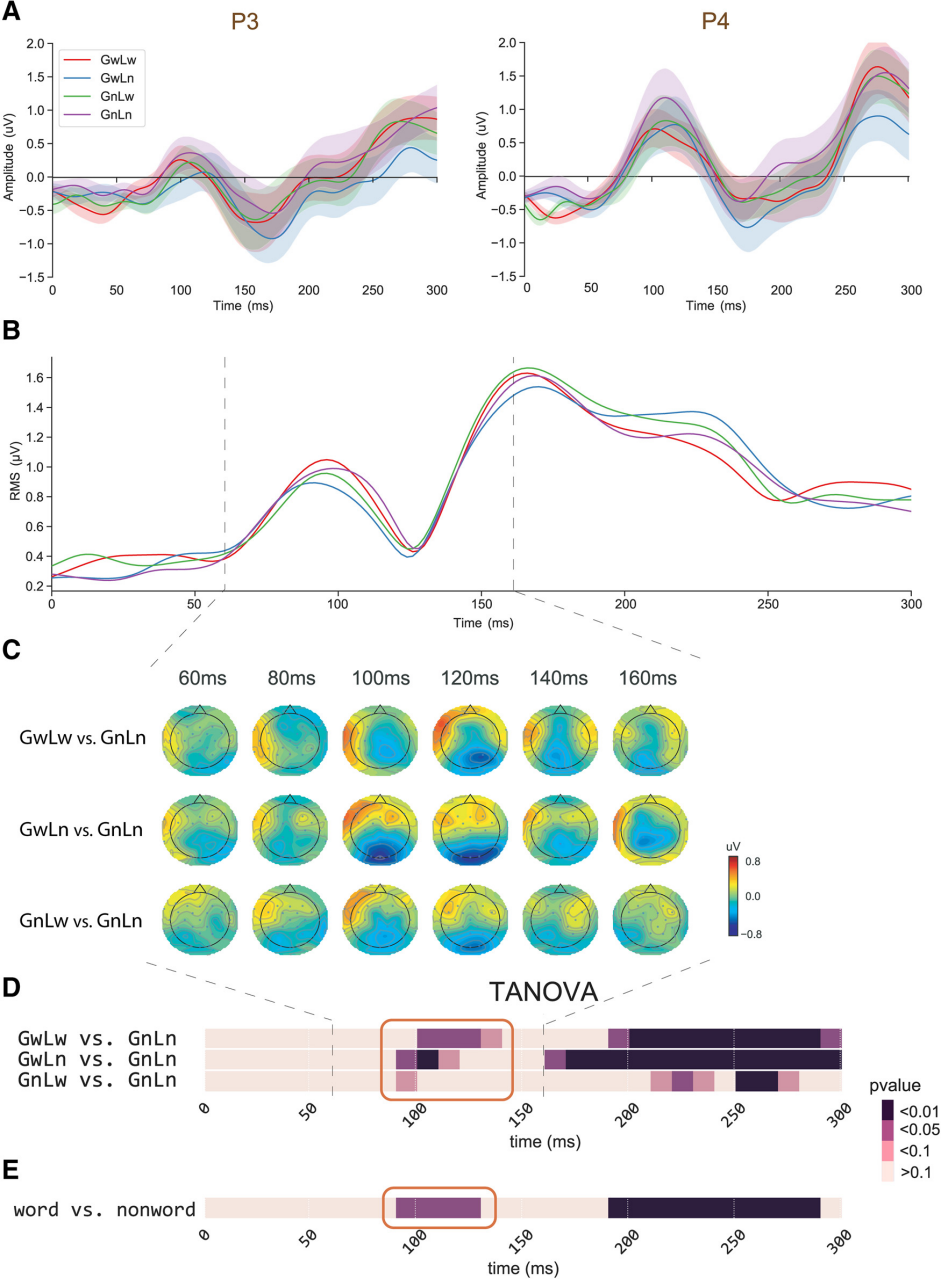
The effects of lexicalized chunks revealed in the paired comparisons between *GnLn* condition and the other three lexical conditions (*GwLw*, *GwLn*, and *GnLw*). ***A***, ERP waveform responses in the representative channels P3 and P4. One-way ANOVA did not reveal any response amplitude differences among conditions. ***B***, RMS waveform responses of all channels. No amplitude difference was found. ***C***, Topographical comparisons of response amplitude. Each row shows a comparison across time. The color scheme depicts the differences in response amplitude between conditions. No significant difference was found on the electrodes after the multiple comparison correction (FDR). ***D***, The temporal dynamics of TANOVA on paired comparisons (uncorrected). The red boxes highlight the earliest latency when the significant differences were observed. All three conditions show evidence of early lexical detection. ***E***, The temporal dynamics of TANOVA on the comparison between *GnLn* and the average of three lexical conditions, corrected by temporal clustering analysis with a corrected threshold of 0.05 (Maris and Oostenveld, 2007). The lexicality effects emerge around 100 ms.

To test the hypothesis about the lexical detection in the earliest stage, we conducted analyses to investigate the lexicality effects at the global and local chunk levels. First, we applied repeated measures one-way ANOVA on the ERPs in P3 and P4, as well as RMS waveforms calculated using all channels (Fig. 3*A*,*B*). We did not find any significant amplitude differences among the four conditions. Next, compared with the responses to *GnLn* strings that contained no lexical chunks at either level, the topographical pattern of response amplitudes in conditions that include lexical chunks (*GwLw*, *GwLn*, and *GnLw*) did not show any significant differences in any sensors after multiple comparison correction (Fig. 3*C*). However, the difference topographies showed distinctive patterns of amplitude distribution (90–130 ms, higher on the left frontal area and lower on occipital area). These results suggest that lexical detection could induce changes in the configuration of neural sources, rather than in response amplitude. Therefore, we investigated the topographic patterns to infer the different configurations of neural processing across conditions.

The analysis of TANOVA revealed significant differences between the topographies of *GnLn* and response patterns in *GwLw*, *GwLn*, and *GnLw* conditions (Fig. 3*D*, highlighted in the red box). The differences were first observed at 90 ms after stimulus onset. The differences were most substantial in the *GwLn-GnLn* comparison as the significant level at *p* < 0.01 for the following 20 ms. The pattern differences were also observed in the *GwLw-GnLn* comparison, but the significant differences started later at 100 ms and lasted for 30 ms. The *p* value of the 90- to 100-ms time bin in the *GnLw-GnLn* comparison was 0.0539, and the relative Bayes factor (BF10) was 2.3371, which indicates weak evidence in favor of H1 (Held and Ott, 2018). The TANOVA of the comparison between *GnLn* and the average of the other three lexical conditions showed significant differences around 110 ms (Fig. 3*E*, highlighted in the red box). TANOVA revealed that topographic differences occurred before 130 ms between lexicalized chunks at either level and the non-lexicalized *GnLn* condition. These results suggested that the early-stage process related to the detection of lexicality. The global level information may facilitate the detection because the effect size ranked from biggest to smallest in the order of *GwLn*, *GwLw*, and *GnLw*.

We also observed significant differences in later responses. The *GwLn* has a significant cluster starting at 150 ms, followed by *GwLw* that has a significant cluster starting around 190 ms. The *GnLw* does not have a significant late cluster until ∼230 ms. These latency differences at a later stage suggested that the lexical processes could first occur at the global level. We further investigate these dynamics in the next session.

#### Processing of chunks at different levels

**Figure 4.**
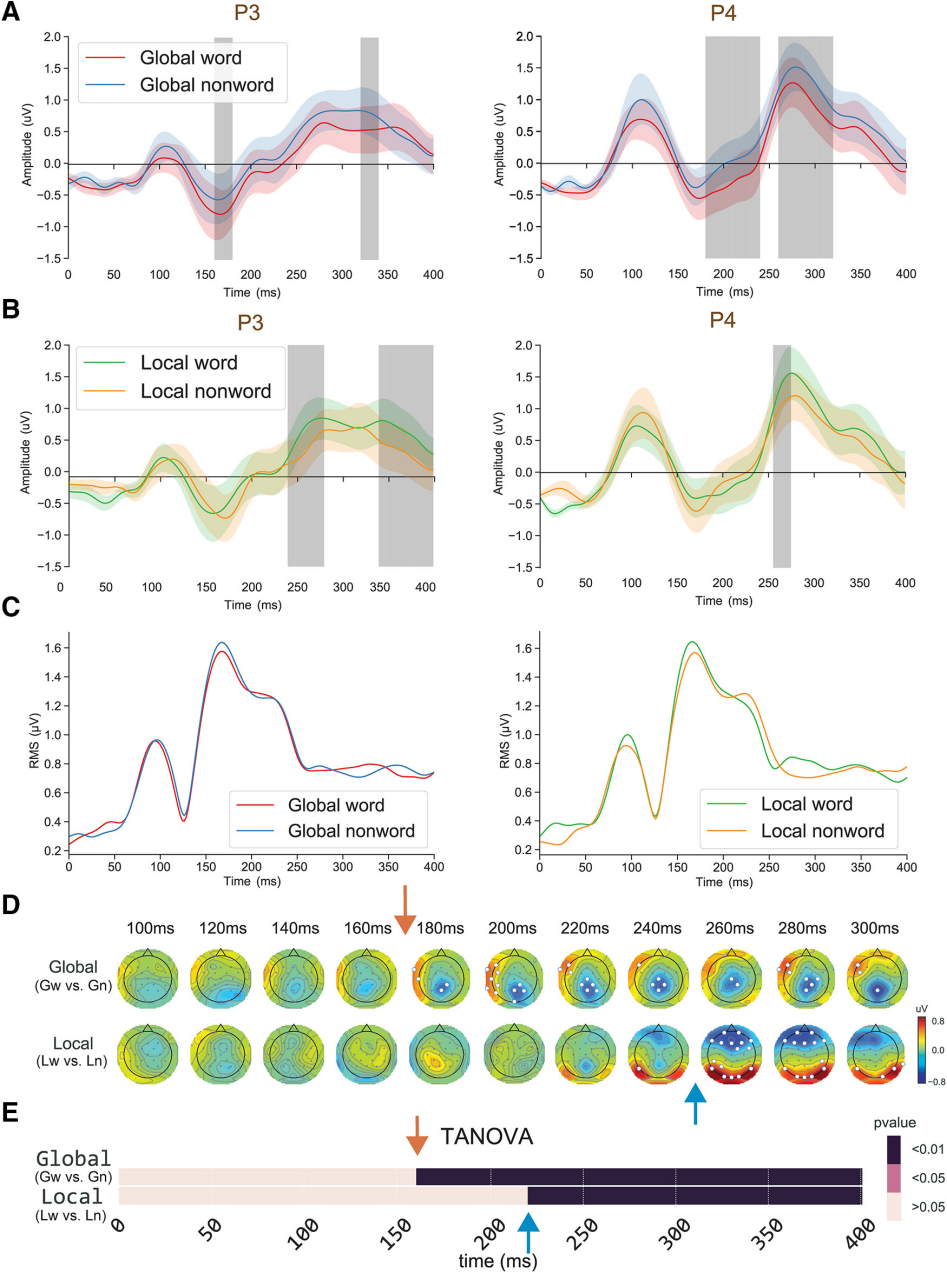
The processing dynamics of chunks at global and local levels. ***A***, ERP waveform responses of the global lexicality effect in the representative channels P3 and P4; *t* tests revealed the effects occurred around 160 ms. The shaded area indicates *p *<* *0.05. ***B***, ERP waveform responses of local lexicality effect in the representative channels P3 and P4. The effects occurred around 250 ms, later than the global lexicality effect in ***A***. The shaded area indicates *p *<* *0.05. ***C***, RMS waveform responses of all channels on global lexicality and local lexicality comparisons. No amplitude difference was found. ***D***, Analysis of response amplitude in topographical comparisons between different lexical status at the global level (upper row) and at the local level (lower row) across time. The color scheme depicts the differences in response amplitude between conditions, and the white points superimposed on the topographies indicate the electrodes that showed significant differences after multiple comparison correction (FDR). ***E***, The temporal dynamics of TANOVA results. The results showed a distinct starting time of significant response pattern differences between different lexical status at the global and local levels. The red arrows in all plots indicate the earliest latency of difference in the global level comparison, and the green arrows indicate the earliest latency of difference in the local level comparison. The results were corrected by a temporal clustering analysis with a corrected threshold of 0.05 (Maris and Oostenveld, 2007).

We applied responses amplitude and pattern analyses between conditions with different lexical status either at the global level or at the local level to test the dynamics of chunk processing. We compared ERPs from the channels of P3 and P4 for words and nonwords, separately at the global and local levels (Fig. 4*A*,*B*), as well as RMS from all channels (Fig. 4*C*). Paired *t* tests were applied to these data. We found that the lexicality effects occurred as early as 160 ms at the global level, but much later around 250 ms at the local level. These effects are in selected parietal channels but are absent in the RMS, suggesting the effects are narrowly distributed, which is consistent with the distribution results in Figure 4*D*. When comparing the strings that contained a global-level word (*GwLw*, *GwLn*) with the strings that contained global-level nonword (*GnLw*, *GnLn*), the significant differences were observed in the electrodes over the middle parietal and left frontal-temporal regions (Fig. 4*D*, indicated by white points) starting around 170 ms (Fig. 4*D*, red arrow). When comparing the strings that contained a local-level word (*GwLw*, *GnLw*) with the strings that contained local-level nonword (*GwLn*, *GnLn)*, the significant differences in response amplitudes started much later at the latency around 230 ms in the electrodes over frontal and parietal-occipital regions (Fig. 4*D*, blue arrow).

The TANOVA results in Figure 4*E* further showed that response patterns were statistically significantly different between processing the distinct lexical status at the global level began around 160 ms after the stimulus onset (Fig. 4*E*, red arrow), and the significant pattern differences at the local level began around 220 ms (Fig. 4*E*, blue arrow), consistent with the observation in Figure 4*A*,*D*, as well as the observations of late differences in Figure 3*D*.

## Discussion

This study investigated the processing dynamics of written texts that included different levels of chunks, such as word and phrase. With the stimuli of Chinese four-character strings that contain multiple grain-size language chunks, the behavioral results showed that the recognition of lexicalized local chunks was affected by the lexical status of global chunks, but not vice versa. These results suggested that the processing of chunks at the global level was prioritized over the processing of local ones during reading. Moreover, the earliest EEG responses showed distinct patterns between lexicalized and non-lexicalized chunks, and the latency of successive EEG responses was faster when processing chunks at the global level than that for local chunks. These consistent behavioral and electrophysiological results suggested that two distinct stages successively operate in the early stage of reading for the detection of potential chunks and further processes on the detected chunks at multiple levels.

### Detection of chunks at 100 ms

In the clustering results (Fig. 2), a “temporal gap” was observed in the early EEG reading responses and separated the processing from 80 to 200 ms into two distinct clusters, suggesting the different neural bases and possible distinct functions. Furthermore, the response patterns of the earliest cluster around 100 ms were modulated by the lexical status of chunks at both global and local levels (Fig. 3). These findings are consistent with the early lexical familiarity checking mechanism proposed in the E-Z reader model (Reichle et al., 2003). Language chunks and their lexical status should be checked before accessing the semantics. In other words, the familiar lexical chunks are detected before subsequent processes (e.g., semantic retrieval). This is especially important in the language that lacks explicit boundaries for lexicalized chunks/phrases, such as written Chinese. Our results suggest such lexical checking/detection can occur early in the reading process around 100 ms and extend to multiple chunk levels.

What factor enables this early chunk detection in reading? Top-down mechanisms have been proposed to account for perceptual and cognitive functions, such as the prior knowledge or prediction of the global shape information in object recognition (Bar, 2003; Bar et al., 2006; Panichello et al., 2012). The detection of language chunks at multiple levels during reading involved the left frontal regions and occipital regions (Fig. 3*A*), similar to the top-down modulation by the early feed-forward projection of low spatial frequency information (Bar et al., 2006). In previous research, high-frequency words can be easily detected and recognized (Monsell and Besner, 1991; Ellis, 2002). The transparency (MacGregor and Shtyrov, 2013) and decomposability (Abel, 2003; Vannest et al., 2005) also affect the mental encoding of complex words, phrases, and idioms. However, individual differences in reading may make the perception of these physical attributes vary across individuals. Therefore, the factor that leads to the early chunk detection is likely to be the perceptual consequences, the familiarity of these attributes. The familiarity has been demonstrated in improving language retrieval (Bannard and Matthews, 2008; Zheng et al., 2015). In this study, we controlled the familiarity by only using stimuli that were rated at the extreme degree of familiarity, either very familiar words or strange nonwords. We speculate that the familiarity of lexical-orthographic features (such as frequency and decomposability) is the criterion of chunk detection, and it can apply simultaneously at both global and local levels during early reading processes.

### The priority of processing global chunks

Our behavioral results demonstrated that the processing of a global chunk significantly affected the lexical decision of lexicalized local chunks. In contrast, the local chunks had no impact on the lexical decision of the lexicalized global chunk. The unidirectional effect suggested that the processing of global level chunks had priority over the processing of their constituents. EEG further provided evidence supporting the temporal hierarchy in processing global and local chunks. The EEG results showed that the processing of global chunks started around 160 ms, while the onset of local chunk processing was much later (∼220 ms). These EEG results, together with our behavioral data, demonstrated that after the simultaneous chunk detection at both levels, the processing of different sizes of lexical chunks began at different times: the processing of global chunks preceded that of local chunks.

The priority of global information has been demonstrated in many cognitive domains. Gestaltism (Heider, 1977; Dewey, 2018) considers the global contains more information than the aggregation of its locals. In vision, the global precedence effect (Navon, 1977/7) suggests that recognizing a scene is hierarchical and global processing has priority over local processing. In contrast, local processing is subject to the top-down reevaluation and integration into global processing. Similarly, the top-down facilitation of visual object recognition also implies that the activation of high-level information will be faster than the lower-level information (Bar, 2003; Bar et al., 2006). In linguistics, the word superiority effect (Reicher, 1969), an advantage of words on recognizing letters, suggests that the processing of a word at the global level interacts with the letter identification (McClelland and Rumelhart, 1981b). This study further demonstrates the influences of phrases on words. Our results expand previous research and suggest that the global-priority mechanism can be applied across multiple levels in a hierarchical manner in the linguistic context. The priority of global chunks is consistent with the information theory (Shannon, 1948): larger chunks contain more context information and less internal entropy, which can prevent ambiguity.

### Paralleled processing of chunks at both levels

The behavioral results revealed that the judgment of a non-lexicalized phrase at the global level was more difficult when the task-unrelated chunks were familiar words at the local level. These results indicated that local processing might be initiated before the finish of global processing. The EEG results further supported that processing at both levels temporally overlapped, the response patterns of processing global chunks continued after the start of local processing response patterns (Fig. 4). This observation of partially temporal overlap in the processing part-whole hierarchies is consistent with simultaneous processing mechanisms implemented in the connectionist networks (Hinton, 1990). A scheduler could control the participation of processing at different levels. Should processing a chunk exceed expected duration, the processing of chunks at other levels would occur. Moreover, the topographic patterns showed left lateralization for processing chunks at the global level, whereas both hemispheres engaged in processing chunks at the local level (Fig. 4), suggesting the possible anatomic differences that mediate the partially temporal paralleled processes at both levels.

### Chunking in a broader cognitive perspective

Various cognitive functions can exert a top-down influence on early perceptual responses. For example, attention is one of the most common functions that modulate early perceptual responses, such as increasing the response gain in the visual (Fries et al., 2001), auditory (Poghosyan and Ioannides, 2008), and somatosensory (Steinmetz et al., 2000) domains. The current study offers a new top-down influence in a linguistic context. The lexicality/accessibility of the character combination determines the way of chunking and recombination of characters to form representations at both global and local scales. Such reconstruction of representations may modulate the early visual responses in reading.

The top-down influence provides a common framework that links among cognitive systems. For example, orofacial motion alters speech perception, such as the McGurk effect (McGurk and MacDonald, 1976), and shortens latency of early auditory responses (Van Wassenhove et al., 2005). Speech articulation dampens the auditory responses to speech feedback (Houde et al., 2002) and modulates the sensitivity to auditory stimuli via the motor-to-sensory transformation (Tian and Poeppel, 2010, 2013, 2015; Tian et al., 2016, 2018; Ma and Tian, 2019; Li et al., 2020). The current study provides evidence supporting that the language system can penetrate and influence visual processing.

Chunking, which deducts combinatory representations into more basic linguistic units for processing, plays a crucial role in language comprehension. Previous studies suggest that linguistic chunking arguably occurs in complex morphology such as decomposing compounds into morphemes – the smallest linguistic unit that carries meaning (Stockall and Marantz, 2006; Fiorentino and Poeppel, 2007). The current study further demonstrates that phrases can be segmented into smaller linguistic units based on lexicality at both global and local levels. Our results bridge chunking in morphology with chunking in sentences based on semantics and syntax (Ding et al., 2016), and even higher linguistic levels such as paragraphs or an entire text based on formal structures and conceptual flow (Teng et al., 2020). A complete picture of chunking operation across all levels of linguistic hierarchy emerges.

The two-stage processing suggested by our results may contribute to the debate regarding the accessible units in complex words (Giraudo and Dal Maso, 2016). Some studies suggest that the morphologic decomposition occurs only on the semantically transparent morphologic pairs (e.g., hunter–HUNT; Meunier and Longtin, 2007). In contrast, other studies found the semantics-opaque but morphology-complex words (e.g., corner = corn + er) also showed decomposition (Davis, 2004; Devlin et al., 2004; Gold and Rastle, 2007). That is, the surface morpheme-like unit that could be an interface between form and meaning is accessible regardless of the semantic relation between the global level and its constituents (Devlin et al., 2004; Gold and Rastle, 2007). Our results are more consistent with the latter view and suggest that this surface morpheme-like unit could be detected automatically as long as it is available. Specifically, these results show that bi-character words, which are bi-morpheme units, are also automatically decomposed from phrases, suggesting that the surface morpheme-like unit in the decomposition is not limited to the basic linguistic morphemes. Furthermore, the access of the surface morpheme-like unit has been localized over the occipito-temporal and left inferior frontal regions (Devlin et al., 2004; Gold and Rastle, 2007; Meinzer et al., 2009; Pliatsikas et al., 2014), which is consistent with our EEG topographic pattern (Fig. 3*C*). Orthographic typicality and lexicality modulate reading responses around 100 ms (Hauk et al., 2006; Faísca et al., 2019), which is also consistent with the detection timing in our observations.

The “global first” principle in different levels of accessible units has been observed in morphology (Bybee, 1995), letter detection (Han et al., 2003), and general vision (Chen, 1982; Wang et al., 2007). All these results are consistent with our findings that processing global level information possesses priority (Fig. 4). Last, the discrepancy between global and local information affects ERP responses as early as 250 ms (Han et al., 1999), suggesting a possible initiation time of parallel processing that is consistent with our results (Fig. 4).

The chunking operation is universal among sensory modalities for processing information that is beyond cognitive capacity. However, the nature of stimuli among sensory modalities may differentiate the possible neural mechanisms that mediate chunking. For example, linguistic information unfolds over time in speech, whereas the information can be available at the same time in reading (e.g., visual field and reading span). Therefore, temporal processing such as neural oscillations might be a potential dominant mechanism for chunking in the auditory domain with neural entrainment to acoustic features (such as prosodic cues and speech envelope; Luo and Poeppel, 2007), top-down rhythmic and melodic template (Nozaradan et al., 2011; Di Liberto et al., 2020), semantic and syntactic cues (Ding et al., 2016, 2017), as well as structures and formats of language (Teng et al., 2020). However, in the visual domain, additional spatial information can be available at a time. Chunking is more likely based on the template from higher hierarchy, such as orthographic template in global/local letters (Kimchi, 1992) and mental representation of lexicality in the current study.

**Figure 5.**
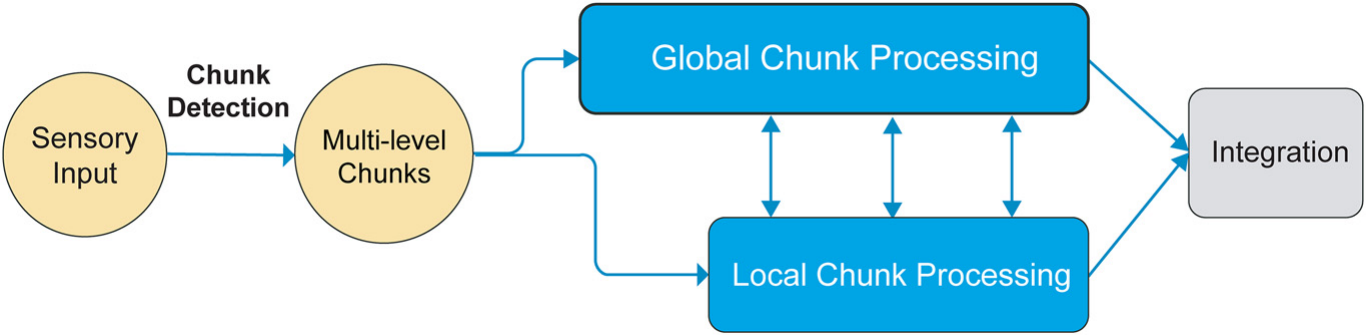
Schematic diagram of proposed two-stage chunking operation in reading.

Based on all results, we tentatively put forward a workflow of processing multiple-level information in reading (Fig. 5). The segmentation occurs in an early short time window, and possible chunks at all levels are detected based on the familiarity of lexical-orthographic features (detection stage). The chunks at each level are further processed with distinct temporal characteristics (processing stage). Specifically, the processing of global chunks possesses priority over the local chunks, while the processing of local chunks can launch before the finish of global chunk processing. Hence, the processes of chunks at two levels have a partially temporal overlap that enables interaction across levels before final integration.

Because our primary goal was to test the relation between lexicality and chunking at different levels, we controlled the lexical-orthographic features such as the number of strokes, and frequencies. Theoretically, lexical access arguably occurs earlier than the semantic process. It is more likely that lexical factors are the primary factors mediating the effects that we observed. Semantic attributes could be another factor influencing the late process of chunking. It would be of interest to study semantics in chunking and obtain a complete understanding. Moreover, we investigated the computational dynamics of chunking in reading by testing the response latencies. EEG is one of the optimal tools to test the dynamics and latency, but not an optimal tool for inferring the spatial location of sources. The spatial distribution in topography is a distorted and incomplete representation of underlying neural sources because the topography is most likely a manifestation of a mixture from multiple neural sources. To avoid confusion, we only take advantage of changes in topographies across time or across conditions to infer the neural dynamics (Tian and Huber, 2008; Tian et al., 2011; Yang et al., 2018; Wang et al., 2019). Nevertheless, the location of the chunking operation is another aspect of interest. We planned to use fMRI for further investigation.

### Conclusion

The current study investigated the chunking mechanism in reading. Consistent behavioral and EEG results suggested that multiple levels of chunks were realized via two distinct stages of chunking in the early time course of reading. The first stage detected lexicalized chunks at all levels of grain-size. In the second stage, in contrast, the processing at the global level led the local level and resulted later in a parallel and interactive process. This study revealed the rich dynamics of chunking operation during reading, which provides the starting computation for comprehension of hierarchical language systems.

## Synthesis

Reviewing Editor: Philippe Tobler, University of Zurich Decisions are customarily a result of the Reviewing Editor and the peer reviewers coming together and discussing their recommendations until a consensus is reached. When revisions are invited, a fact-based synthesis statement explaining their decision and outlining what is needed to prepare a revision will be listed below. The following reviewer(s) agreed to reveal their identity: Jed Meltzer, Antje Strauss.

Dear Dr. Tian

Thank you for submitting your paper to eNeuro. The reviewers found the study to be carefully designed and to have the potential to make a useful contribution to the literature. However, they raised several important points, each of which needs to be carefully addressed in a revisions of the paper.

The current study shows that the presence of different chunk sizes in reading has an impact on rather early stages of cortical processing starting around 100 ms after stimulus onset. More precisely, the current data suggests different neural generators of the N100 depending on chunk sizes. It speaks to the timely debate on entrainment at different linguistic level (e.g. words and phrases) and whether this is just reflecting regularly elicited N100s. The current data would speak in favor of this view. Moreover, the current results suggest that there is another way of top-down influence to the N100 other than attention, which is rather linguistic in nature.

Major points

1. It is hard to interpret the results as clearly pointing to two levels of visual information processing in the model presented by the authors, especially in the absence of any comparison to previous literature about ERP components involved in reading that exhibit lexicality effects. There have been many studies comparing words vs. nonwords, which have identified specific ERP components that are modulated by lexicality in specific time windows. The present results should be compared to such studies. Do either the global or local effects match previous literature? Presumably there have also been studies of familiar vs. unfamiliar compound words or phrases, which may relate to the global lexicality concept explored in this study.

2. On a related note, to achieve its full potential for to the field of auditory cognitive neuroscience, the paper should engage with and discuss the ongoing debate on speech segmentation or chunking.

A few partly random selections of references to this debate include:

Ding, N., Melloni, L., Zhang, H. et al. Cortical tracking of hierarchical linguistic structures in connected speech. Nat Neurosci 19, 158-164 (2016) doi:10.1038/nn.4186

Ding N, Melloni L, Yang A, Wang Y, Zhang W and Poeppel D (2017) Characterizing Neural Entrainment to Hierarchical Linguistic Units using Electroencephalography (EEG). Front. Hum. Neurosci. 11:481. doi: 10.3389/fnhum.2017.00481

Nozaradan S, Peretz I, Missal M and Mouraux A (2011). Tagging the Neuronal Entrainment to Beat and Meter. J Neurosci, 31 (28) 10234-10240; DOI: https://doi.org/10.1523/JNEUROSCI.0411-11.2011

3. The current results suggest that there is another way of top-down influence to the N100 other than attention, which is rather linguistic in nature, which would be important to stress. While processing of visual and auditory information is different, a crosstalk between fields might be very important to further our understanding.

4. Did the Authors control for semantic factors as well? One could imagine that lexicalized compound words (GwLw & GwLn) might be more abstract or would score less when asking participants to rate imagebility. They might be semantically richer, delivering more information to process?

5. The presentation of the ERP data is extremely limited, with no displays of the sensor signals in different conditions, but only the very abstract display of difference topographies and the output of the TANOVA clustering algorithm. This also makes it very difficult to compare this study to previous studies that compared word vs. nonword stimuli. The clearest way to view ERP data is to see the average waveform in different conditions, allowing the reader to evaluate differences in both amplitude and latency of response components. As a result, the study shows that the brain distinguishes between the 4 conditions used in the experiment, but the reader is not left with a clear view of whether the results support a 2-stage model of global vs. local processing, and how these results relate to established measures of lexicality.

6. Methods - Stimuli (p. 7): There are also studies on complex morphology showing that derivatives like compounds need more processing effort. For (psycho-)linguists it would be important to relate the computational term of “chunking” to one of the more classical linguistic terms. What is happening, when we are “chunking”?

7. Statistics - The way how they are reported is problematic. A p-value greater than 0.05 is not significant. Maybe one could say there is a trend, but the description as “marginally significant” is not objective. Hence, the conclusion (on p. 14 line 311&312: “the chunk at global level influenced lexical decision more than chunks at local level”) is not justified by the statistics for the moment. In order to conclude that, effect sizes and Bayesian statistics (for example by using JASP, a freeware available here: https://jasp-stats.org/) for all computed statistics are necessary.

Intermediate and minor issues:

1. On p. 15 line 334, the authors need to be more careful when phrasing their conclusions. Absent evidence is not evidence for absence. It might be best to simply skip the second part of the sentence “suggesting the lexical status of local chunks... etc.”.

2. On p. 19 lines 432 to 433: in order to conclude that differences are “weaker", effect sizes are needed. However, it is bizarre that the Authors even mention “a difference” here, this is opposite to their remark in the caption to Figure 3B, where they state that “the scale p<0.1 was chosen only for visual inspection”. The authors should include again Bayesian statistics here, because this way they can be sure, whether they have found “weak” or “anecdotal” evidence or whether there is actually no evidence speaking in favor of H1 but rather in favor of H0.

3. Table 1. Please add phonetic transcriptions (Pinyin or equivalent if not Mandarin) of the Chinese words in the examples. It makes it easier for a non-Chinese reader to look them up in a dictionary if they are curious.

4. GwLn condition - most readers of this papers will not be familiar with the concept of the 4-character Chinese idioms known as “Chengyu,” and the fact that the existence of these in the Chinese language makes this experiment possible. Most readers will therefore find this paper hard to follow. For a non-Chinese reader, it is difficult to understand how two 2-character nonwords can add up to a 4-character “word.” The authors should add more explicit explanation about Chengyu for the reader to understand the nature of these expressions. Specifically, how they consist of 4 separate 1-character morphemes (or words).

5. P. 11 - “Compared with TANOVA” - what is TANOVA? It hasn’t been mentioned before. Is it necessary to discuss it when introducing the current method of EEG multivariate analysis? If so, it needs to be explained.

6. On page 13, the authors do introduce TANOVA - now one is confused about whether the cluster analysis “compared with TANOVA” is an additional method used in parallel, or is somehow part of the TANOVA process.

7. Relatedly, further confusion arises during the report of the TANOVA when comparing Fig. 2B with the description on p. 13 lines 297--301i. It seems as if clusters are determined topographically with flexible durations. Shouldn’t be the time axis basically thrown away in the statistical comparisons? However, in Fig. 3B and 4B it seems as if the authors have binned the data in time and the time bins are not constrained by the cluster extent in each condition.

8. P. 13 - How does a cluster size of 2 continuous samples overcome the multiple comparison problem? It seems like an arbitrary number. Usually temporal clustering involves an empirical estimation of the family-wise error rate or false discovery rate based on cluster size, as in the widely used method of Maris et al. implemented in FieldTrip. The authors need to justify their choice of 2 as the cluster size.

9. P. 11 - averaged EEG “data across all participants to get ERPs at each time point for each group; b) defined ERPs at each time point in each group as a sample,” What is meant by “group” here? This experiment does not seem to involve multiple groups.

10. “The reaction times were not different between the trials with underlines either below the 350 first or the last two characters (p=0.44), suggesting the positions of stimuli that were relevant to task did not affect response speed.” Apparently, half the trials involved an underline under all 4 characters, and half under only 2, but position of the underline was not listed as a factor in the ANOVA, so how was this tested? Is it possible that position could have interacted with the other factors even without a main effect? It seems like it should be included in the ANOVA, although they can still produce the same figures and post-hoc tests in the absence of main and interaction effects with the position factor.

11. Fig 2 - There is an inconsistency with the main text; the figure legend says that “Cluster 2nd” appears earlier in the symbol condition, with shorter duration; the main text describes “Cluster 3rd.” Overall it is extremely difficult to tell which cluster is which in that figure - Please use a wider variety of colours as the chosen colours are all very similar. There is no need for these colours to be on a quantitative scale as they are simply labels. It would also be helpful for the reader if color scales in 2B and 3B/4B are different, because they depict very different things.

12. Relatedly, Fig. 2B: In order for the reader to better understand the clusters that have been found by the algorithm, and to get an idea about why/on which basis these clusters have been identified, it would be nice to actually show topographies and time series of each cluster.

13. Fig. 3A: The authors need to be very careful here, because they did not find any differences between conditions when comparing amplitudes. The red box should be removed.

14. For clarification: In Fig. 3B, it is clearly seen that there are also late differences that were not followed-up and are not discussed any further. Why?

15. Overall the cluster analysis presented in Figure 2 is not very informative, and does not have much impact on the hypotheses of the study other than to show that processing 4-character strings is very different than processing 1-symbol stimuli. Please either say why it is important to have this analysis or remove it.In the last TANOVA analysis, where results are reported in Fig. 4, one is missing the test for the interaction. Is there a reason why only the two main effects global (GwLw/GwLn vs. GnLw/GnLn) and local (GwLw/ GnLw vs. GwLn/GnLn) have been tested? One would think that GnLn as a higher-level baseline behaves really different than the other three conditions, which could be only seen when testing the interaction.

16. It is a bit surprising that in the discussion, the authors mainly focus on the timing, stating that processing global chunks happens earlier than processing local chunks. However, the analysis of the classical N1-P2 amplitudes had not revealed any differences - presumably also not the N1-P2 latencies, right? This was the reason why the TANOVA has been chosen, which is blurring the timing. In any case, should the interpretation of the results not focus more on the different brain areas that are apparently contributing to the generation of the different topographies in each condition? To this end, it could be also helpful to compute the second-order derivative (the surface Laplacian) of the EEG potential distribution, namely the scalp current density, to get a better idea about the sources (source analysis with only 32 electrodes is certainly not very meaningful).

17. On p. 5, the authors report that 21 participants had been recruited. On p. 10 it is reported that 5 participants have been excluded due to extensive artefacts. This information should be mentioned on p. 5.

18. p.11 line 245: typo: “driving” should be “driven"

19. Whenever the Authors report absolute differences in reaction times, no units are mentioned, probably ms, e.g., p.14 line 324 and line 327 but also on the following pages.

20. p. 15 Lines 337-340: this explanation is too dense and not easy to parse. Maybe the authors can take more time here to explain the rationale of the EEG-Experiment.

21. p.23 line 529: typo: “speculated” should be “speculate"
